# Overexpression of *PagSTOMAGEN*, a Positive Regulator of Stomatal Density, Promotes Vegetative Growth in Poplar

**DOI:** 10.3390/ijms231710165

**Published:** 2022-09-05

**Authors:** Yufei Xia, Kang Du, Aoyu Ling, Wenqi Wu, Jiang Li, Xiangyang Kang

**Affiliations:** 1 National Engineering Research Center of Tree Breeding and Ecological Remediation, Beijing Forestry University, Beijing 100083, China; 2 Key Laboratory of Genetics and Breeding in Forest Trees and Ornamental Plants, Ministry of Education, College of Biological Sciences and Technology, Beijing Forestry University, Beijing 100083, China; 3 Institute of Crop Sciences, Chinese Academy of Agricultural Sciences, Beijing 100081, China

**Keywords:** *PagSTOMAGEN*, stomatal density, photosynthesis, plant hormone, vegetative growth, poplar

## Abstract

Poplar is an important fast-growing tree, and its photosynthetic capacity directly affects its vegetative growth. Stomatal density is closely related to photosynthetic capacity and growth characteristics in plants. Here, we isolated *PagSTOMAGEN* from the hybrid poplar (*Populus alba × Populus glandulosa*) clone 84K and investigated its biological function in vegetative growth. *PagSTOMAGEN* was expressed predominantly in young tissues and localized in the plasma membrane. Compared with wild-type 84K poplars, *PagSTOMAGEN*-overexpressing plants displayed an increased plant height, leaf area, internode number, basal diameter, biomass, IAA content, IPR content, and stomatal density. Higher stomatal density improved the net photosynthetic rate, stomatal conductance, intercellular CO_2_ concentration, and transpiration rate in transgenic poplar. The differential expression of genes related to stomatal development showed a diverged influence of *PagSTOMAGEN* at different stages of stomatal development. Finally, transcriptomic analysis showed that *PagSTOMAGEN* affected vegetative growth by affecting the expression of photosynthesis and plant hormone-related genes (such as *SAUR75*, *PQL2*, *PSBX*, *ERF1*, *GNC*, *GRF5*, and *ARF11*). Taken together, our data indicate that *PagSTOMAGEN* could positively regulate stomatal density and increase the photosynthetic rate and plant hormone content, thereby promoting vegetative growth in poplar. Our study is of great significance for understanding the relationship between stoma, photosynthesis, and yield breeding in poplar.

## 1. Introduction

Stomata, composed of a pair of guard cells, are channels on the surface of plant leaves [1,2] which regulate gas exchange between plants and the atmosphere and play an important role in maintaining plant photosynthesis and water-use efficiency [3,4,5,6]. The formation of mature stomata undergoes several intermediate steps, termed the stomatal lineage, from meristematic cells (MMC) to stomatal lineage ground cells (SLGC), guard cell mother cells (GMC), and finally, the formation of guard cells (GC) [7,8,9]. During stomatal development, stomata are specified and positioned nonrandomly through asymmetric cell division and the integration of intercellular signaling [10].

Stomatal density is regulated by positive and negative genetic factors [11,12]. Negative regulators include the cell surface receptor *TOO MANY MOUTHS* (*TMM*) [13], *ERECTA* family receptor-like kinases (*ER*, *ERL1*, and *ERL2*) [14] and their ligands *EPF1* and *EPF2* [15,16,17], as well as *STOMATAL DENSITY AND DISTRIBUTION 1* (*SDD1*) functioning in a *TMM*-dependent manner [18,19]. Positive regulators include the three core *bHLH* transcriptional regulators *SPEECHLESS*, *MUTE*, and *FAMA* [20,21,22], and *STOMAGEN/EPFL9* (*STOMAGEN*) belonging to the *EPF/EPFL* family [23].

Some genes have pleiotropic functions. Stomatal density regulators have been reported to affect plant growth and development or stress resistance. For example, *EPF1* of the *EPF/EPFL* family not only regulates stomatal development, but also affects plant biomass in *Arabidopsis* [24]. *EPFL6* of the *EPF/EPFL* family affects stomatal density and drought resistance in poplar [25]. However, *STOMAGEN*, which is also a member of the *EPF/EPFL* family, has only been reported to affect the stomatal development of plants, but it has not been found to affect the biomass of plants [26]. *STOMAGEN* encodes a cysteine-rich precursor that is a signaling factor for stomatal differentiation [26,27,28]. *STOMAGEN* overexpression can increase stomatal density in Arabidopsis, which improves the photosynthetic rate and transpiration rate. However, vegetative growth is not affected because the reduced water-use efficiency causes water stress in overexpressing plants [23,26,27].

Vegetative growth is very important for poplar [29]. The vegetative growth of plants is affected by many factors, such as photosynthesis [30], plant hormones [31], environmental factors [31], etc. Photosynthesis is a prerequisite for biomass production [30], and is affected by a large number of key genes, such as the photosystem key factors *PSAB* [32], *PSAN* [33], and *PSBX* [34], as well as some important growth regulators, such as *GRF5* [35], *GNC* [36], and *PIFs* [37]. Auxin-responsive factors, such as *ARFs* and *SAUR*-like auxin-responsive protein [38,39,40], are all differentially expressed when stimulated by auxin. Cytokinins are mainly affected by *CKX* family genes [41]. The differential expression of these genes is the reason for the difference in the vegetative growth of different plants.

As an important woody plant, it is unclear whether stomatal density and vegetative growth are regulated by *STOMAGEN* in poplar. Thus, we identified and cloned *PagSTOMAGEN* from ‘84K’ poplar. Phylogeny and sequence analysis showed that *PagSTOMAGEN* was homologous with *STOMAGENs* from *Arabidopsis thaliana*, *Populus trichocarpa*, and *rice* [42]. The overexpression of *PagSTOMAGEN* in 84K poplar increased stomatal density, which further affected photosynthetic capacity, enzymatic activity, plant hormone content, and biomass. Moreover, differentially expressed genes (DEGs) related to stomatal development in overexpressing poplar showed that *PagSTOMAGEN* was involved in various stages of stomatal development [43]. Based on these experimental data, we preliminarily explored the biological function of *PagSTOMAGEN*, which provided theoretical support for high yield by increasing the stomatal density in poplar.

## 2. Results

### 2.1. Isolation and Identification of PagSTOMAGEN

**Figure 1:**
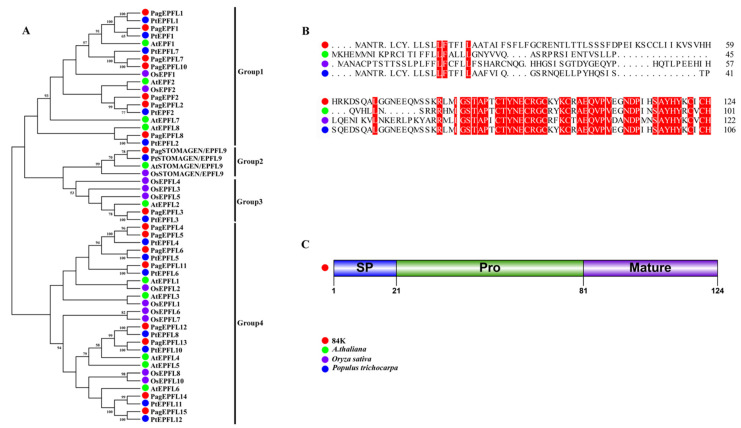
Isolation and identification of *PagSTOMAGEN*. (**A**) Phylogenetic tree of EPF/EPFL gene family. The bootstrap values are shown. (**B**) Amino acid sequence alignment of STOMAGEN/EPFL9 proteins in 84K, *Arabidopsis thaliana*, *Oryza sativa*, and *Populus trichocarpa*. Identical amino acids are highlighted in red. (**C**) Schematic representation of the domain structure of *PagSTOMAGEN/EPFL9*. *PagSTOMAGEN/EPFL9* is composed of the signal peptide (SP), the propeptide (Pro), and the mature domain. Amino acid positions are indicated by numbers.

To illustrate the phylogeny and sequence characteristics of *STOMAGEN* in poplar, the EPF/EPFL gene family was retrieved from the 84K protein database using *Arabidopsis* AtEPF/EPFL proteins as queries. An unrooted neighbor-joining tree was constructed using 54 full-length protein sequences from four plant species (Figure 1A, Table S2). The results show that *PagSTOMAGEN/EPFL9* was highly homologous with *STOMAGEN/EPFL9s* from other plants, which was further confirmed by amino acid sequence alignment and three-dimensional structural models (Figure 1B and Figure S1).

The *PagSTOMAGEN/EPFL9* gene encodes a small polypeptide of 124 amino acids with a putative signal peptide (SP) at the N-terminus (Figure 1C). The mature form of the *PagSTOMAGEN/EPFL9* gene product, stomagen, is a peptide consisting of the C-terminal 43 amino acids of the precursor protein. It is probable that the *PagSTOMAGEN/EPFL9* peptide is also subject to post-translational processing, which needs to be further determined in 84K.

### 2.2. Expression Patterns and Subcellular Localizations of PagSTOMAGEN

**Figure 2:**
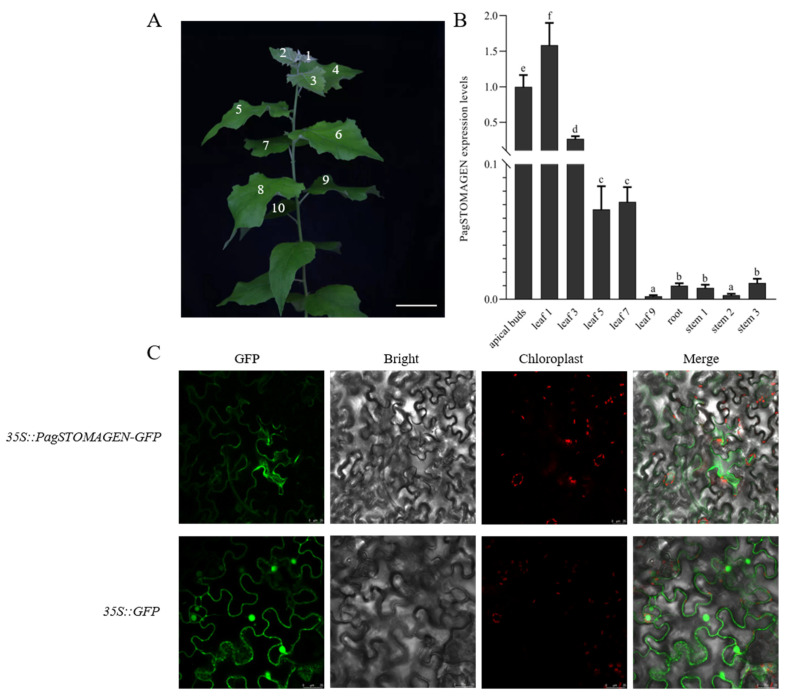
Expression patterns and subcellular protein localization of *PagSTOMAGEN*. (**A**) Definition of leaf numbers. Bar = 5 cm. (**B**) Expression patterns of *PagSTOMAGEN* in different tissues. *PagACTIN* was used as a reference. Error bars represent the standard deviations (SD) of three biological replicates. *p*  < 0.05 was considered statistically significant and is represented by different letters. (**C**) Subcellular localization of the *PagSTOMAGEN* protein. Laser confocal microscopy was used to obtain images of *tobacco* leaves with transient expression of *35S::PagSTOMAGEN-GFP* and *35S::GFP*. Bars = 25 μm.

To preliminarily explore the biological roles of *PagSTOMAGEN*, the expression of *PagSTOMAGEN* was detected in the apical buds, in the first, third, fifth, seventh, and ninth leaf blades, in stems from the second to fifth internodes, and in the roots using RT-qPCR. Significant differential expression patterns were observed (Figure 2A,B). *PagSTOMAGEN* showed high expression in apical the buds and young leaves; its expression was low among the other tissues analyzed (Figure 2B). Therefore, *PagSTOMAGEN* may play a significant role in apical buds and young leaves.

To evaluate the subcellular localization of *PagSTOMAGEN*, *35S::PagSTOMAGEN-GFP* and *35S::GFP* constructs were generated and transiently expressed in *Nicotiana benthamiana*. Fluorescence from *35S::PagSTOMAGEN-GFP* was detected only on the plasma membrane (Figure 2C), while *35S::GFP* fluorescence was detected on the cell membrane and in the nucleus (Figure 2C). The results show that *PagSTOMAGEN* might function on the plasma membrane.

### 2.3. PagSTOMAGEN Positively Regulates the Vegetative Growth of Poplar

**Figure 3:**
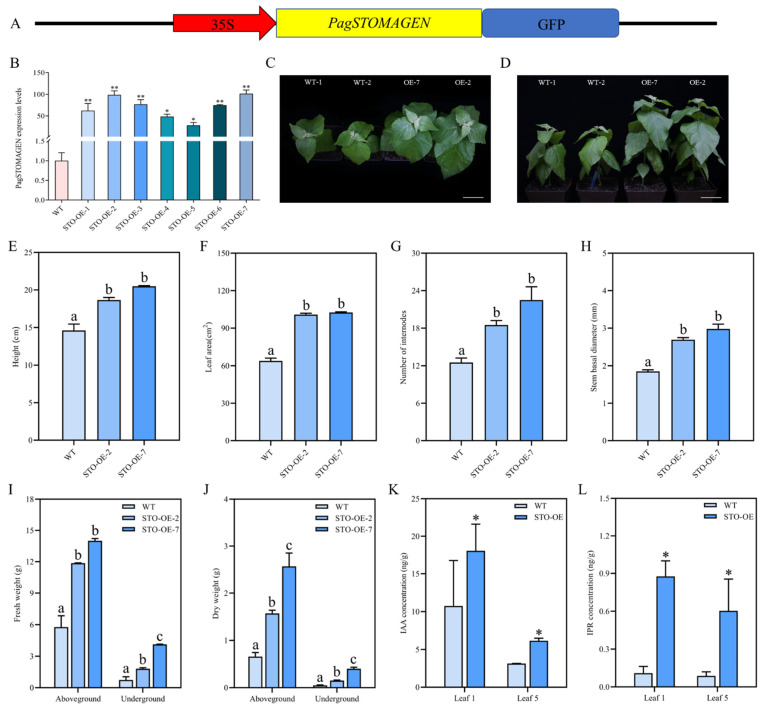
Generation and phenotypic changes in *PagSTOMAGEN*-overexpressing (STO-OE) lines. (**A**) Schematic diagram of the *PagSTOMAGEN* overexpression vector. (**B**) Expression levels of *PagSTOMAGEN* in the STO-OE lines. Values represent the mean ± SD (n = 5). (**C**,**D**) Two-month-old plants. WT: non-transgenic control; OE: *PagSTOMAGEN*-overexpressing lines. Bars = 5 cm. (**E**) Measurement of plant height. (**F**) Measurement of leaf area. (**G**) Measurement of the number of internodes. (**H**) Measurement of stem basal diameter. (**I**) Aboveground and underground fresh weight of STO-OE and WT lines. (**J**) Aboveground and underground dry weight of STO-OE and WT lines. (**K**) IAA concentration in different leaf positions of STO-OE and WT plants. (**L**) IPR concentration in different leaf positions of STO-OE and WT plants. Error bars represent the SD for each genotype with nine plants. Asterisks indicate significant differences in the Student’s *t*-test. ** *p* < 0.01; * *p* < 0.05. *p* < 0.05 was considered statistically significant and is represented by different letters.

To further elucidate the biological function of *PagSTOMAGEN* in poplar, STO-OE lines were generated (Figure 3A–D and Figure S2). All the STO-OE lines showed similar phenotypes (Figure S3), of which STO-OE-2 and STO-OE-7 had the highest *PagSTOMAGEN* expression levels and were selected for further analysis (Figure 3B–D). We investigated the phenotypes of the STO-OE and wild-type (WT) lines using two-month-old plants grown in the greenhouse. Compared with the WT plants, the plant height, leaf area, number of internodes, and stem diameter were significantly increased in the STO-OE lines (Figure 3E–H). Our investigation showed increases in plant height of 27.8 and 40.9%, in leaf area of 58.2 and 80%, in number of internodes of 48 and 60.8%, and stem diameter of 45.4 and 61.1%, respectively, in STO-OE plants compared to the WT (Figure 3E–H). These growth changes indicate that STO-OE might have a higher biomass than the WT.

To determine whether biomass was increased in STO-OE, whole STO-OE and WT plants were divided into aboveground and underground parts, and their fresh and dry weights were measured (Figure 3I,J). The results show that the aboveground and underground biomass of STO-OE were both larger than those of the WT. The aboveground fresh weight of STO-OE-2 and STO-OE-7 was 2.06 and 2.43 times higher than that of the WT, respectively (Figure 3I). The underground fresh weight of STO-OE-2 and STO-OE-7 was 2.46 and 5.61 times higher than that of the WT, respectively (Figure 3I). At the same time, the dry weight of the aboveground parts of STO-OE-2 and STO-OE-7 was 2.39 and 3.90 times higher than that of the WT (Figure 3J). The underground dry weight of STO-OE-2 and STO-OE-7 was 2.97 and 7.92 times higher than that of the WT, respectively (Figure 3J).

In order to explore the reasons for the increase in plant biomass after the overexpression of *PagSTOMAGEN*, we measured the contents of auxin IAA and cytokinin IPR in STO-OE and WT plants. The results show that the IAA content of STO-OE plants was 1.68 and 1.96 times higher than that of the WT at the first and fifth leaf positions, respectively (Figure 3K). At the first and fifth leaf positions, the IPR content of STO-OE plants was 8.04 and 6.85 times higher than that of the WT, respectively (Figure 3L).

### 2.4. Involvement of PagSTOMAGEN in Stomatal Density, Size, and Stomatal Index

**Figure 4:**
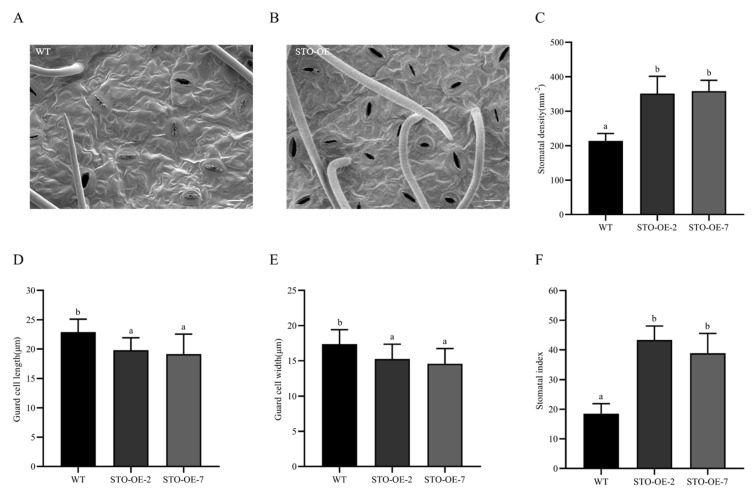
Stomatal density, size, and stomatal index in STO-OE and WT poplar. (**A**) Abaxial leaf epidermis of WT lines. (**B**) Abaxial leaf epidermis of STO-OE lines. (**C**) Stomatal density of STO-OE and WT lines. (**D**) Guard cell length of STO-OE and WT lines. (**E**) Guard cell width of STO-OE and WT lines. (**F**) Stomatal index of STO-OE and WT lines. Error bars are indicated by the SD of five biological replicates. *p*  <  0.05 was considered significantly different and is shown by different letters. Bars = 20 μm.

To determine whether *PagSTOMAGEN* was involved in the regulation of stomatal development as *Arabidopsis*, stomatal density and size were detected in the STO-OE lines. STO-OE showed higher stomatal density, ranging from 351 to 358 mm^−2^, compared to 213 mm^−2^ in WT plants (Figure 4A–C). However, the stomatal size of STO-OE was significantly smaller than that of the WT (Figure 4D,E). The stomatal index and the number of stomata showed similar variations to stomatal density in the STO-OE lines (Figure 4F and Figure S4). In summary, our results suggest that *PagSTOMAGEN* was involved in the regulation of stomatal density, size, and stomatal index.

### 2.5. PagSTOMAGEN Enhances Photosynthetic Capacity by Increasing Stomatal Density

**Figure 5:**
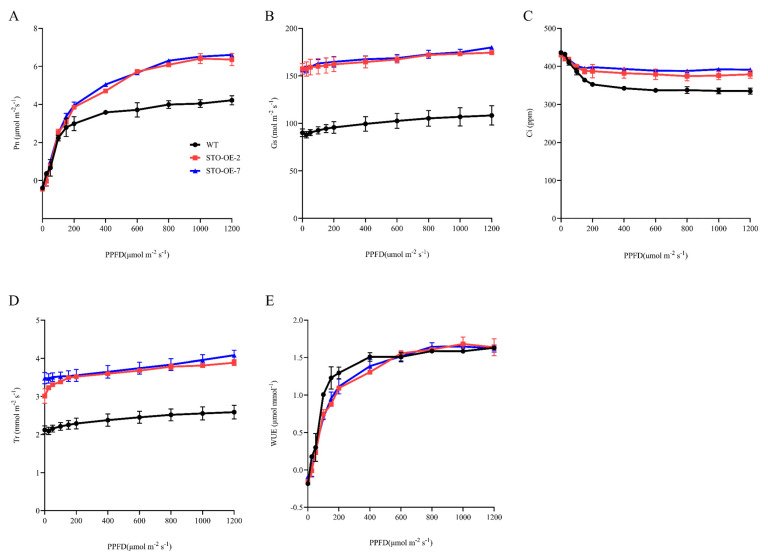
Overexpression of *PagSTOMAGEN* enhances photosynthesis in poplar. Light-response curves were obtained for STO-OE and WT. (**A**) Net photosynthetic rate (*Pn*)–light curve. (**B**) Stomatal conductance (*Gs*)–light curve. (**C**) Intracellular CO_2_ concentration (*Ci*)–light curve. (**D**) Transpiration rate (*Tr*)–light curve. (**E**) Water-use efficiency (*WUE*)–light curve. Error bars are indicated by the SD of three biological replicates.

To determine whether stomatal density is involved in plant photosynthesis, we measured the light-response curves of two-month-old WT, STO-OE-2, and STO-OE-7 plants. The net photosynthetic rate (*Pn*), stomatal conductance (*Gs*), intracellular CO_2_ concentration (*Ci*), and transpiration rate (*Tr*) were significantly higher in STO-OE than in the WT (Figure 5A–D), which agreed with the stomatal density changes (Figure 4A–C). However, there was no significant difference in water-use efficiency (*WUE*) between the WT and STO-OE plants (Figure 5E).

### 2.6. Influence of PagSTOMAGEN on Enzyme Activity and Malondialdehyde Content

**Figure 6:**
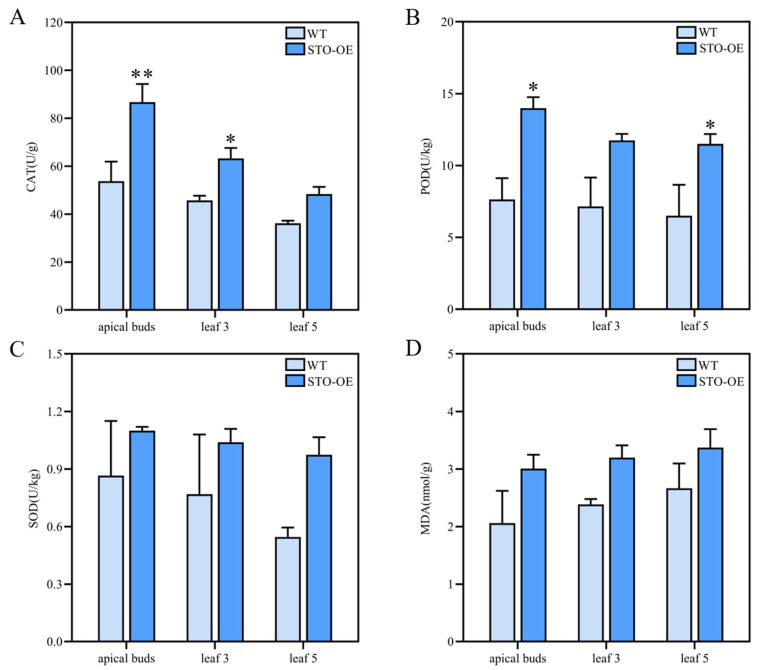
Enzyme activities and malondialdehyde content of STO-OE and WT poplar. (**A**) Activity of catalase (CAT) in STO-OE and WT lines. (**B**) Activity of peroxidase (POD) in STO-OE and WT lines. (**C**) Activity of superoxide dismutase (SOD) in STO-OE and WT lines. (**D**) Malondialdehyde (MDA) content of STO-OE and WT lines. Values are the mean ± SD (n > 3). Asterisks denote significant differences: ** *p* < 0.01; * *p* < 0.05.

Due to the involvement of CAT, POD, and SOD activities and MDA content in plant growth, these physiological indexes were determined. The CAT activity of STO-OE was significantly higher than that of the WT in apical buds and the third leaf position, while a significant difference was not observed in the fifth leaf position (Figure 6A). POD activity was significantly higher in STO-OE in the apical buds and the fifth leaf position, but there was no significant difference in the third leaf position (Figure 6B). SOD activity and MDA content exhibited no significant differences in any of the detected tissues between the STO-OE and WT lines (Figure 6C,D). Taken together, *PagSTOMAGEN* overexpression increased CAT and POD activities but had a little influence on SOD activity and MDA content.

### 2.7. PagSTOMAGEN Modulates the Expression of Genes Related to Stomatal Development and Patterning

**Figure 7:**
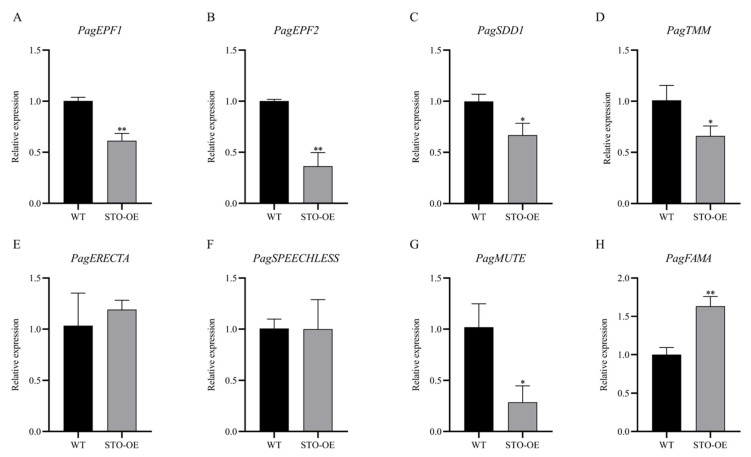
*PagSTOMAGEN* affects the expression of genes involved in stomatal development and patterning. (**A**) Relative expression level of *PagEPF1* in STO-OE and WT lines. (**B**) Relative expression level of *PagEPF2* in STO-OE and WT lines. (**C**) Relative expression level of *PagSDD1* in STO-OE and WT lines. (**D**) Relative expression level of *PagTMM* in STO-OE and WT lines. (**E**) Relative expression level of *PagERECTA* in STO-OE and WT lines. (**F**) Relative expression level of *PagSPEECHLESS* in STO-OE and WT lines. (**G**) Relative expression level of *PagMUTE* in STO-OE and WT lines. (**H**) Relative expression level of *PagFAMA* in STO-OE and WT lines. Expression was estimated using RT-qPCR normalized to *PagACTIN* expression. Error bars represent the SD (data are the means of three biological replicates). Asterisks indicate significant differences in the Student’s *t*-test. ** *p* < 0.01; * *p* < 0.05.

In *Arabidopsis*, *AtSTOMAGEN* affects the expression of genes involved in stomatal development and patterning. To determine whether *PagSTOMAGEN* modulates genes related to stomatal development and patterning in poplar, the expression of these genes was examined in the STO-OE and WT lines. The expression levels of *PagEPF1* and *PagEPF2*, two negative regulators of stomatal formation, were significantly reduced in the STO-OE lines (Figure 7A,B). The expression of the stomatal density inhibitor *PagSDD1* was also significantly decreased (Figure 7C). *PagTMM* and *PagERECTA*, two putative receptors of *PagSTOMAGEN*, showed decreased and unchanged expression, respectively, in STO-OE poplar (Figure 7D,E). Three *bHLH* transcription factors, *PagSPEECHLESS*, *PagFAMA*, and *PagMUTE*, exhibited diverse expression patterns in STO-OE. No changes were observed for the expression of *PagSPEECHLESS* (Figure 7F), which is involved in the first step of stomatal development by regulating the differentiation of protodermal cells. *PagSTOMAGEN* overexpression decreased the expression of *PagMUTE* (Figure 7G). The expression level of *PagFAMA* was significantly increased by *PagSTOMAGEN* overexpression (Figure 7H).

### 2.8. PagSTOMAGEN Promotes the Accumulation of Plant Biomass by Positively Regulating the Expression of Photosynthesis and Growth-Related Genes

**Figure 8:**
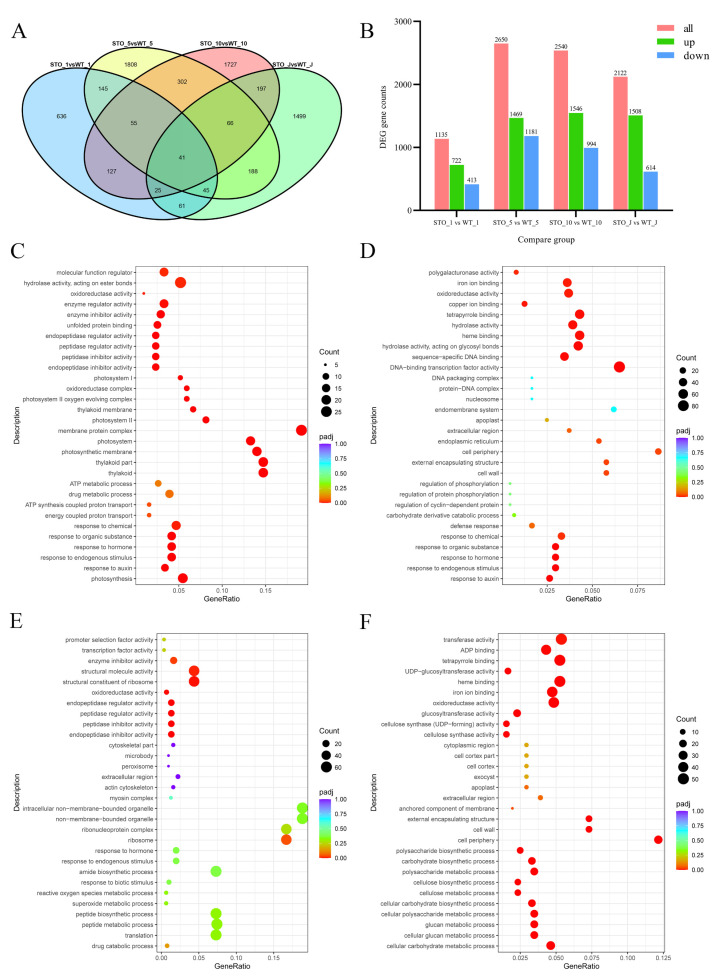
Identification of DEGs in *PagSTOMAGEN*-OE 84K poplar transgenic lines and GO enrichment analysis. (**A**) Venn plots for different sample combinations. (**B**) DEG gene counts for different sample combinations. Includes all up-regulated and down-regulated gene counts. GO enrichment analysis of 1st leaf position (**C**), 5th leaf position (**D**), 10th leaf position (**E**), and young stem (**F**) in STO-OE and WT lines. The Y-axis indicates the GO pathway; the X-axis indicates the gene ratio. The dot size indicates the number of DEGs in the pathway, and the dot color indicates the *p*-value.

The phenotypic changes, particularly the higher biomass in STO-OE lines, prompted us to identify DEGs whose transcription was changed in the leaves and stems. To achieve this, we ran RNA-seq experiments with three STO-OE lines (*35S::PagSTOMAGEN-GFP-nos*) and three WT 84K poplar replicates. We compared transgenic lines to WT for DEG identification (Figure 3B, Table S3). We identified 1135 DEGs (corrected *p* < 0.05) by comparing the transgenic and WT 84K poplar lines in the first leaf position (Figure 8A,B, Table S4). Of these DEGs, 722 were up-regulated while 413 were down-regulated (Figure 8A,B, Table S4). In the fifth leaf position, 2650 DEGs were identified, with 1469 genes up-regulated and 1181 down-regulated, respectively (Figure 8A,B). In the 10th leaf position, there were 2540 DEGs, of which 1546 genes were up-regulated and 994 genes were down-regulated (Figure 8A,B, Table S4). In young stem segments, there were 2122 DEGs, of which 1508 genes were up-regulated and 614 genes were down-regulated (Figure 8A,B, Table S4).

The Gene Ontology (GO) enrichment of differential genes in different tissues was not the same. In the first leaf position, differential genes were mainly enriched in pathways such as photosynthesis, the response to auxin, and the photosystem (Figure 8C, Table S5). In the fifth leaf position, differential genes were mainly enriched in response to auxin, the response to hormones, and cell wall pathways (Figure 8D, Table S6). In the 10th leaf position, differential genes were mainly enriched in pathways such as the response to hormones, transcription factor activity, and structural molecule activity (Figure 8E, Table S7). In young stem segments, differential genes were mainly enriched in the cellular carbohydrate metabolic process, the cellular carbohydrate biosynthetic process, and cell wall pathways (Figure 8F, Table S8).

**Figure 9:**
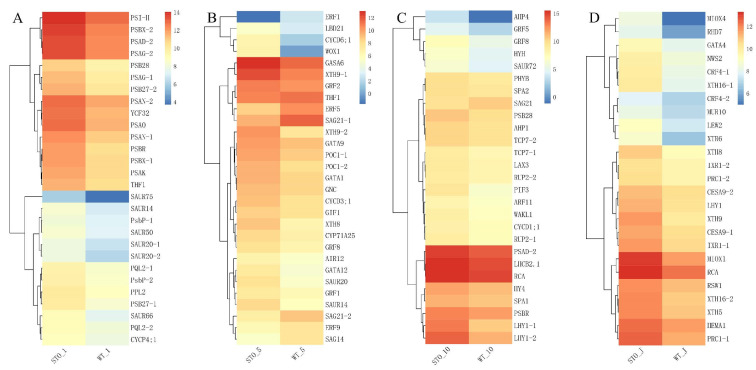
The analysis of changes in DEGs involved in photosynthesis and growth. (**A**) Heatmap of DEGs of STO-OE and WT lines in 1st leaf position. (**B**) Heatmap of DEGs of STO-OE and WT lines in 5th leaf position. (**C**) Heatmap of DEGs of STO-OE and WT lines in 10th leaf position. (**D**) Heatmap of DEGs of STO-OE and WT lines in young stem. The color scale represents the FPKM value.

Differential genes between the WT and STO-OE lines were enriched in photosynthesis and hormone-related pathways in different tissues. In the first leaf position, the auxin-responsive genes *SAUR75*, *SAUR20*, *SAUR66*, *SAUR14*, *SAUR50*, etc. were significantly up-regulated in STO-OE plants (Figure 9A, Table S9). At the same time, the expression levels of photosystem-related genes such as *PQL2*, *PSBX*, *PSAG,* and *PSAN* in overexpressed plants were significantly higher than those in WT (Figure 9A, Table S9). These results may be the reason the overexpressed plants have stronger photosynthesis than the WT, thereby promoting biomass accumulation (Figure 9A, Figure 3). In the fifth leaf position, the expression levels of ethylene-responsive factors *ERF1*, *ERF5*, and *ERF9* and the senescence-related genes *SAG14*, *SAG21*, etc. in STO-OE were significantly lower than those in the WT, while the expression levels of the growth regulators *GNC*, *GRF8*, *GRF2*, *GRF1*, etc. were significantly higher than those in the WT, suggesting that these genes may be responsible for the growth advantage of overexpressed plants (Figure 9B, Table S10). The expression levels of the auxin-responsive genes *SAUR20* and *SAUR14* in the overexpressed plants at this leaf position were significantly higher than those in the WT, which further confirmed that the auxin content of the overexpressed plants was higher than that of the WT (Figure 9B, Figure 3K). At the same time, the expression levels of the cyclins *CYCD3;1* and *CYCD6;1* in the overexpressed *PagSTOMAGEN* plants were also significantly higher than those in the WT. This shows that the high expression of these genes in the fifth leaf position promoted cell proliferation and differentiation, making the overexpressed plants appear bigger leaf size (Figure 9B, Figure 3F). In the 10th leaf position, the growth regulators (*GRF8* and *GRF5*) and the auxin response factor (*ARF11*) were significantly up-regulated in the overexpressed plants (Figure 9C, Table S11). The senescence-associated gene *SAG21* was significantly down-regulated (Figure 9C, Table S11). In the young stem segment, the internode near the terminal bud, the cell wall expansion-related gene *XTH9* was significantly up-regulated in overexpressing plants, which promoted cell expansion (Figure 9D, Table S12).

We performed a correlation analysis between *STOMAGEN* and some differentially expressed genes related to growth, and then, constructed a co-expression network. The pearson correlation coefficient was used to evaluate the gene pair’s expression correlation. The screening threshold for high correlation was *r* > 0.85 and *p* < 0.05. The larger nodes had stronger connectivity degrees, indicating that the genes may be more important. We identified 314 pairs correlated with *STOMAGEN* and 27 genes in this co-expression network (Figure S5). The results show that the reason *STOMAGEN* overexpression in poplar affected plant photosynthesis and biomass might be that it affected the expression of a large number of photosynthesis- and growth-related genes. Through correlation analysis, we found that *STOMAGEN* had the highest correlation with photosynthesis-related genes, such as *TMP14*, *PQL2*, *PDE332*, *THF1*, *PSAD*, *PSAN*, and *PSAK* (Figure S5). Among them, *PQL2* functions in the chloroplast NAD (P) H dehydrogenase (NDH) complex to provide additional ATP for CO_2_ assimilation, followed by *HEMA1*, *WOX1*, *PIF4,* and other genes with strong correlations (Figure S5). These genes are closely related to plant growth and can directly or indirectly promote the biomass accumulation of plants, which is also the reason for the increased biomass in STO-OE poplar, which promotes the vegetative growth of poplar. By analyzing the transcriptome data of *PagSTOMAGEN*-overexpressing plants, we showed that *PagSTOMAGEN* altered the expression of genes related to photosynthesis and plant hormone response, which, in turn, promoted poplar growth and development.

## 3. Discussion

### 3.1. Overexpression of PagSTOMAGEN Increases Stomatal Density in Poplar

This study was the first to demonstrate, in woody plants, that the overexpression of *PagSTOMAGEN* could increase the stomatal density of poplar by 64.8–68.1% (Figure 4A–C). Although this was consistent with the results in *Arabidopsis*, we found that the mechanism by which *PagSTOMAGEN* affected stomatal development in poplar was different to that in *Arabidopsis*.

The mature form of *STOMAGEN* contains 45 amino acids and positively regulates stomatal density in *Arabidopsis* [23,27,44], while *EPF1* and *EPF2* negatively regulate stomatal density [42,45,46,47]. In this study, *PagSTOMAGEN* from 84K poplar was homologous with *STOMAGEN* in *Arabidopsis*, *rice*, and *Populus trichocarpa* (Figure 1A,B), and its mature form contained 43 amino acids (Figure 1C). Since stomata are mainly formed in young tissues, the tissue-specific expression patterns of *PagSTOMAGEN* mainly being expressed in apical buds and young leaves showed that *PagSTOMAGEN* may directly regulate stomatal formation (Figure 2A,B).

The three *bHLH* family transcription factors—*SPEECHLESS*, *MUTE*, and *FAMA*—are involved in the sequential regulation of the stomatal lineage [7,20,48]. Previous studies have demonstrated that *STOMAGEN* acts in the early stage of stomatal formation, which is the MMC to the SLGC stage [49]. *STOMAGEN* acts in the early stage of stomatal development by inhibiting the expression of its receptors to trigger the *MAPK* cascade, which, in turn, inhibits the expression of *SPEECHLESS* [49]. We detected the expression levels of stomatal development-related genes using RT-qPCR and found that three *bHLH* transcription factors showed inconsistent expression changes in STO-OE plants. The expression level of *PagSPEECHLESS* was basically unchanged; the expression level of *PagFAMA*, which functions in the final step of stomatal formation [11], was significantly increased; and the expression level of *PagMUTE*, which limits the frequency of asymmetric division and promotes the differentiation of meristemoids to GMCs [50], was significantly reduced (Figure 7F–H). Therefore, we speculated that *PagSTOMAGEN* played an important role in the middle and later stages of stomatal development in poplar, which is from the SLGC to the GC stage. This was a new finding based on previous research results.

At the same time, we also found that *PagSTOMAGEN* can inhibit the expression of the stomatal density suppressors *PagEPF1* and *PagEPF2* (Figure 7A,B), which indicates that *PagSTOMAGEN* could improve stomatal density by reducing the expression of its antagonists in poplar. A similar trend was observed for the *PagSDD1* gene (Figure 7C), which encodes a stomatal density suppressor [18,51,52,53]. The expression of its receptor *PagTMM* was also inhibited (Figure 7D). It has been shown that *PagSTOMAGEN* increases stomatal density because it competes with *PagEPF1* and *PagEPF2* to bind to the receptor-like protein *PagTMM* and inhibit the expression of *PagTMM* [44,54]. However, the expression of *PagERECTA*, which is also a receptor for *PagSTOMAGEN*, did not change significantly between WT and *PagSTOMAGEN*-overexpressing plants (Figure 7E). It was speculated that there may be a certain competition between *PagERECTA* and *PagTMM*, which resulted in the combination of less *PagSTOMAGEN* with *PagERECTA*. The transcriptional changes in these genes suggest that *PagSTOMAGEN* was directly or indirectly involved in the diverse stages of the stomatal lineage.

### 3.2. Overexpression of PagSTOMAGEN Promotes the Vegetative Growth of Poplar

Poplar is one of the most widely planted tree species in the world due to its important greening function and economic value [55]. It is also an important raw material for paper, construction and the bioenergy industry [56]. STO-OE poplar displayed an increased plant height, leaf area, number of internodes, stem basal diameter, and biomass compared to the WT (Figure 3E–J and Figure S3). This indicates that *PagSTOMAGEN* overexpression can promote vegetative growth in poplar. However, *AtSTOMAGEN* overexpression cannot enhance vegetative growth in *Arabidopsis* under constant light conditions [26]. It was unclear why poplar overexpressing *PagSTOMAGEN* behaved differently to *Arabidopsis* in vegetative growth.

Firstly, we analyzed the reasons for the growth advantage of STO-OE poplar on the phenotypic and physiological levels. *PagSTOMAGEN* overexpression enhanced the photosynthetic rate in poplar. The enhanced photosynthetic rate was caused by increased stomatal conductance and intercellular carbon dioxide concentration in STO-OE plants (Figure 5A–C). This indicates that increased stomatal density enhanced the photosynthetic rate by regulating the diffusion process of CO_2_ gas [26,57,58], which is consistent with *Arabidopsis*.

Although the photosynthetic rate and transpiration rate were enhanced in the STO-OE lines for poplar and *Arabidopsis* (Figure 5A,D), *WUE* displayed diverse changes. In *Arabidopsis*, *WUE* decreased in *AtSTOMAGEN*-overexpressing lines, which subjected the plants to water stress, thereby resulting in no significant change in vegetative growth [26]. In poplar, there was no significant difference in *WUE* and MDA content between the STO-OE and WT lines (Figure 3E and Figure 6D), which indicates that *PagSTOMAGEN*-overexpressing poplar was not subjected to water stress, further enhancing vegetative growth (Figure 3I,J). These results clearly show that increased stomatal density enhanced photosynthetic capacity but had no effects on *WUE* in STO-OE poplar. At the same time, studies have shown that an increase in CAT and POD enzyme activities can promote biomass accumulation [4]. CAT and POD enzyme activities were significantly increased in *PagSTOMAGEN*-overexpressing poplar compared with the WT (Figure 6A,B), which may also explain the vegetative growth advantage in STO-OE poplar.

Then, we explored the reasons for the increase in the biomass of STO-OE poplar at the level of molecular mechanisms. Changes in biomass are affected by many factors. Plant hormones such as auxin and cytokinin play vital roles in regulating the growth and development of poplar, and they control plant height, leaf area, and cell size [35,59]. We assessed the auxin and cytokinin contents of the WT and STO-OE and found that the auxin and cytokinin contents were substantially increased in STO-OE poplar (Figure 3K,L). These results verified that *PagSTOMAGEN* promoted vegetative growth by promoting the accumulation of auxin and cytokinin in poplar.

Through the transcriptome analysis of different tissues of the WT and STO-OE, we found that auxin pathway-related genes were significantly highly expressed in STO-OE at the 1st, 5th, and 10th leaf positions and young stem segments, such as *SAUR75*, *SAUR14*, *SAUR50*, *SAUR20*, *SAUR66*, *SAUR72,* and *ARF11* (Figure 9). *SAUR50* is involved in the de-etiolation of *Arabidopsis* cotyledons [60]. Auxin response factor (*ARF*) family genes play an important role in controlling sensitivity to the plant hormone auxin [61]. This result indicates that different tissues in the STO-OE lines were affected by high auxin content, compared to WT, and auxin response-related genes could regulate auxin sensitivity, thereby promoting poplar growth.

We also compared the expression level of the genes involved in photosynthesis signaling pathways in the leaf and stem of the WT and STO-OE lines. As shown in Figure 9, the expression levels of *PSBX*, *PSAD*, *PSAG*, *PSAN*, *THF1*, *PQL2*, *POC1*, *GIF1*, *GNC*, and *GRF5* in STO-OE were increased compared to those in the WT. *THF1* is involved in the metabolic pathway that controls the assembly of the PS II complex [62]. *GNC*-overexpressing poplars exhibited a higher biomass accumulation, chlorophyll content, photosynthetic rate, and plant height, compared with the WT [36]. *GRF5* significantly enlarged the leaf size in *GRF5*-overexpressing transgenic poplars by enhancing both cell division and cell expansion [35]. These examples demonstrate that the high expression of these transcription factors in the STO-OE lines was responsible for the increase in plant photosynthetic rate and biomass.

At the same time, the expression levels of *ERF1*, *ERF5* and *ERF9* involved in the ethylene pathway were reduced in the STO-OE lines, compared with the WT (Figure 9). The constitutive expression of *ERF1* phenocopies exhibited ethylene over-production [63]. Thus, their low expression inhibited the accumulation of ethylene in STO-OE poplar, thereby delaying plant senescence and prolonging photosynthetically active periods. In addition, the senescence-related transcription factor *SAG14*, which promotes plant senescence [64], was also expressed at a lower level in STO-OE plants than in the WT. These results are consistent with the plant hormone and photosynthetic capacity analyses (Figure 3K and Figure 5A), suggesting that *PagSTOMAGEN* gene expression might affect hormone content and photosynthetic rate and that these changes affected leaf and stem development in poplar.

The above findings clarify that the overexpression of *PagSTOMAGEN* could promote the photosynthetic rate and transpiration rate by increasing stomatal density in poplar. This resulted in increased biomass in poplar. Our study indicates that *PagSTOMAGEN* overexpression had positive effects on the stomatal development and vegetative growth of poplar. Therefore, selecting a gene to improve multiple traits in poplar simultaneously could be an effective strategy. Whether the *PagSTOMAGEN* gene is also involved in the regulation of other important growth and development traits and the pathways that encode the signal effect remains to be explored in the future.

## 4. Materials and Methods

### 4.1. Sequence Retrieval and Gene Identification

The deduced amino acid sequences from *Arabidopsis* AtEPF/EPFL proteins were downloaded from the TAIR database (http://www.arabidopsis.org, accessed on 15 December 2021). *Arabidopsis* AtEPF/EPFL proteins were blast analyzed against the 84K protein sequences using the BLASTp algorithm [65,66]. The protein sequences of EPF/EPFLs from *Oryza sativa* and *Populus trichocarpa* were downloaded from Phytozome (http://phytozome.jgi.doe.gov/pz/portal.html, accessed on 15 December 2021) (Table S2). The neighbor-joining (NJ) tree was constructed via the maximum likelihood method using MEGA 7 with 1000 bootstrap replicates [67]. Multiple sequence alignment was performed for *STOMAGEN/EPFL9* (*STOMAGEN*) from four species using ClustalW (http://www.ebi.ac.uk/Tools/clustalw/, accessed on 15 December 2021) [68]. The protein structure was predicted using I-TASSER (https://zhanggroup.org/I-TASSER/, accessed on 19 December 2021).

### 4.2. Plant Material and Growth Conditions

The poplar line ‘84K’ (*Populus alba × P. glandulosa cv.*) was used for gene cloning, expression pattern analysis, and genetic transformation. The tissue cultures of 84K poplar were grown on 1/2 Murashige and Skoog (MS) agar medium at 25 °C under a photoperiod of 16 h light and 8 h dark [69]. The seeds of *Nicotiana benthamiana* were sown in pots filled with a 3:1 mixture of soil and perlite in a growth chamber at 22 °C with a 16 h/d light photoperiod. They were grown for 30 days, and then, used for subcellular localization assays.

### 4.3. RNA Extraction and Gene Cloning

Total RNA was isolated from the collected plant tissues using a plant total RNA extraction kit (Tiangen, China, Cat DP432) in accordance with the manufacturer’s instructions, and treated with DNase I (Tiangen, China, Cat DP432). The quality and quantity of RNA were measured using a NanoDrop 2000 Spectrophotometer (Thermo Fisher Scientific, Waltham, MA, USA). First-strand cDNA was synthesized from the total RNA using a cDNA Synthesis Kit (Tiangen, China, Cat KR106) as per the manual. The *PagSTOMAGEN* coding sequence was cloned using the PrimeStar^®^ High-fidelity Thermostable DNA Polymerase Reagent Kit (Takara Biotechnology Co., Ltd., Dalian, Liaoning, China), and the primers are shown in Supplementary Table S1.

### 4.4. Quantitative Real-Time Polymerase Chain Reaction Analysis

Total RNA extracted from various poplar tissues was reverse-transcribed into cDNA using a cDNA Synthesis Kit (Tiangen, China, Cat KR106). Quantitative real-time polymerase chain reaction (RT-qPCR) assays were performed using TransStart Top Green qPCR SuperMix (TRANSGEN, China, cat AQ132-22) on the Applied Biosystems 7500 real-time PCR system according to the manufacturer’s manual. Three technical replicates and three biological replicates were performed for each tissue sample. *PagACTIN* was used as an internal control, as described previously [35]. Gene-specific primers were obtained by directly querying the primer data from qPrimerDB (https://biodb.swu.edu.cn/qprimerdb, accessed on 13 December 2021) [70]. The 2^−ΔΔCt^ method was used for calculating gene relative expression levels. The primers are listed in Supplementary Table S1.

### 4.5. Subcellular Localization

The ORF of *PagSTOMAGEN* without the termination codon was PCR-amplified and inserted into *pBI121-GFP* to yield *35S::PagSTOMAGEN-GFP* (Figure 3A), which had a cauliflower mosaic virus 35S promotor upstream of the cloning site. The *35S::PagSTOMAGEN-GFP* and *35S::GFP* were transformed into *Agrobacterium tumefaciens* strain GV3101, and then, injected into the leaf lamina of *Nicotiana benthamiana* plants as described previously [35]. Two days after injection, transformed tobacco leaves were observed using laser confocal fluorescence microscopy (Leica TCS SP8; Leica, Wetzlar, Germany). An argon ion wavelength of 488 nm was employed for GFP and chlorophyll. Fluorescence was detected at 495–515 nm for GFP and at 650 nm for chlorophyll [68].

### 4.6. Plant Transformation and Molecular Identification of Transgenic Poplar

The constructed *35S::PagSTOMAGEN-GFP* was introduced into the *Agrobacterium* strain GV3101 using the freeze–thaw method [71]. The *Agrobacterium tumefaciens*-mediated transformation of 84K poplar was performed as described previously [35]. The infected leaves were transplanted to MS agar medium containing 30 mg∙L^−1^ kanamycin and 200 mg∙L^−1^ timentin for screening. Regenerating buds were transferred to 1/2 MS agar medium with 30 mg∙L^−1^ kanamycin and 200 mg∙L^−1^ timentin until rooting.

Genomic DNA was extracted from each presumptive transgenic line and the WT plant using a Plant Genome Extraction Kit (Tiangen, China, Cat DP320). Transformation was verified using primers the 35S-F and *PagSTOMAGEN*-R (Table S1). The expression level of *PagSTOMAGEN* was detected using RT-qPCR. The 84K transgenic lines identified via PCR and RT-qPCR were used for propagation. One-month-old transgenic plants were transplanted to the greenhouse (16 h light/8 h dark, 22–25 °C, relative humidity: 40–45%).

### 4.7. Evaluation of Plant Growth

The plant height, leaf area, number of internodes, and stem diameter were measured in two-month-old transgenic and WT plants with nine individual plants per line. Aboveground and underground plant materials were collected and weighed to obtain the fresh weight for the WT and *PagSTOMAGEN*-overexpressing (STO-OE) lines. The above materials were dried to a constant weight at 80 °C to obtain their dry weight using the gravimetric method.

### 4.8. Detection of Plant Hormones

The plant samples (leaves) were harvested from the WT and STO-OE plants, frozen in liquid nitrogen, and stored at −80 °C. Following the manufacturer’ s protocols of Convinced (Nanjing Convinced-test Technology Co., Ltd., Nanjing, China), the samples were dissolved in methanol/water/formic acid (15:4:1, *v/v/v*). A total of 10 μL of 100 ng/mL internal standard mixed solution (IS) was added into the extract to allow for quantification. The extract was then evaporated to dryness, dissolved in methanol, and filtered. Auxin and cytokinin were detected using a liquid chromatography-tandem mass spectrometry (LC-MS/MS) system (AB Sciex QTRAP 6500) from Nanjing Convinced-test Technology Co., Ltd. Three biological replicates were analyzed.

### 4.9. Determination of Stomatal Density, Guard Cell Length and Width, and Stomatal Index

Scanning electron microscopy analysis of the leaves was carried out for two-month-old WT and STO-OE lines according to the previous procedure, with minor modifications [72]. The leaves were fixed in 2.5% glutaraldehyde at 4 °C. The tissue blocks were washed with 0.1 M Phosphate Buffer (PB, pH 7.4) three times and transferred to 1% osmium tetroxide (OsO4) made in 0.1 M PB (pH 7.4) for 1–2 h at room temperature. Subsequently, the tissue blocks were washed three times using 0.1 M PB (pH 7.4). The washed tissue blocks were dehydrated in a graded ethanol series and immersed in tert-butanol for 30 min. The samples were dried in the K850 Critical Point Dryer (Quorum Technologies Ltd., Lewes, UK), and then, attached to metallic stubs using carbon stickers and sputter-coated with gold for 30 s. The images were observed and taken with a scanning electron microscope. Stomatal density and guard cell length and width were determined from the images. A stomatal index (SI) was calculated using the following formula: SI = (number of stomata) /(number of stomata + number of other epidermal cells) × 100.

### 4.10. Gas Exchange Analysis

A LI-COR 6400 portable photosynthesis meter was used to measure the net photosynthetic rate (*Pn*), stomatal conductance (*Gs*), intercellular CO_2_ concentration (*Ci*), and transpiration rate (*Tr*) in two-month-old WT and STO-OE plants for 4–5 h after the beginning of the photoperiod. The seventh and eighth leaves were used because they represented mature leaves with complete stomatal development, and their photosynthetic parameters were relatively stable. Photosynthetic light-response curves were determined at photosynthetic photon flux densities (PPFD) of 1200, 1000, 800, 600, 400, 200, 150, 100, 50, 25, and 0 μmol m^−2^⋅s^−1^ with 400 μmol mol^−1^ external CO_2_. *WUE* was calculated as the ratio *Pn*/*Tr*.

### 4.11. Measurement of Catalase, Peroxidase, and Superoxide Dismutase Activities

Catalase (CAT), peroxidase (POD), and superoxide dismutase (SOD) activities were measured according to the manufacturer’s instructions (Nanjing Mofan Biotechnology Co., Ltd., Nanjing, China). Briefly, 0.1 g of leaves from two-month-old plants was ground into fine powder. The powder was added to a 1 mL extract, and then, extracted for 3–5 min in an ice bath using a tissue crusher. The extracted solution was centrifuged at 8000× *g* for 10 min at 4 °C, and the supernatant was collected. The absorbance was measured at 405, 470, and 560 nm for CAT, POD, and SOD, respectively, using a SpectraMax 190 full-wavelength microplate reader (Molecular Devices, Sunnyvale, CA, USA) [73].

### 4.12. Measurement of Malondialdehyde Content

Malondialdehyde (MDA) concentration was measured using the thiobarbituric acid method, with minor modifications [74]. Leaves (0.2 g) from two-month-old plants were homogenized with 3 mL of 10% thiobarbituric acid at 4 °C. The supernatant was collected after centrifugation at 8000× *g* for 10 min at 4 °C. Equal volumes of the supernatant and 0.6% thiobarbituric acid were mixed. The obtained mixture was reacted in a boiling water bath for 20 min, and then, cooled down immediately. The cooled mixture was centrifuged at 4000 rpm for 10 min, and the supernatant was collected. The light absorbance (A) of the supernatant was measured at 450, 532, and 600 nm using an MD SpectraMax 190 full-wavelength microplate reader [75]. MDA content (nmol/g) = MDA concentration × volume of extracted liquid (mL)/fresh weight (g).

### 4.13. Transcriptome Analysis

The 1st, 5th, and 10th leaf positions and the young stems samples used for genome-wide transcriptome sequencing in the WT and STO-OE plants were harvested, and then, quickly frozen in liquid nitrogen. A TRIzol reagent kit (Invitrogen, Carlsbad, CA, USA) was used to extract the total RNA from the samples, and an RNase-Free DNaseSet (Qiagen China, Shanghai, China) was used to purify the RNA. To detect the integrity of the RNA, we performed agarose gel electrophoresis, and a NanoDrop 2000 biological analyzer (Thermo Fisher Scientific Inc., Wilmington, DE, USA) was used to detect the concentration of RNA. High-quality RNA was used for subsequent sequencing. Transcriptome sequencing of the samples was performed by Nuohezhiyuan Technology Co., Ltd. (Beijing, China). Then, the FPKM of each gene was calculated based on the length of the gene and the read counts mapped to this gene. The DESeq R package was used for differential gene expression analysis [76]. Genes for which an adjusted *p*-value ≤ 0.05 was found by DESeq were assigned as differentially expressed. Information on gene annotation was acquired from the NCBI (http://www.ncbi.nlm.nih.gov/), JGI (http://jgi.doe.gov/), PopGenie (http://popgenie.org/), Tair (http://www.arabidopsis.org/), and KEGG (http://www.kegg.jp/) databases.

### 4.14. Statistical Analyses

SPSS software (IBM Corp., Armonk, NY, USA) was used to perform the statistical analyses, and the data are represented as the mean ± SD. The Student’s *t*-test and one-way ANOVA were used to determine variance among the mean values [77].

## Data Availability

The data presented in this study are available in Supplementary Materials.

## Supplementary Materials

The supporting information can be downloaded at: https://www.mdpi.com/article/10.3390/ijms231710165/s1.
