# Supplementary material for: Overexpression of PagSTOMAGEN, a Positive Regulator of Stomatal Density, Promotes Vegetative Growth in Poplar
Source: Int J Mol Sci. 2022 Sep 5;23(17):10165. doi: 10.3390/ijms231710165 (PMC9456429; doi:10.3390/ijms231710165)
Supplement: Supplementary file 1 [file ijms-23-10165-s001.zip › ijms-1892419-supplementary/Supplementary_Materials.pdf]

Supplemental Data

The following supplemental materials are available:

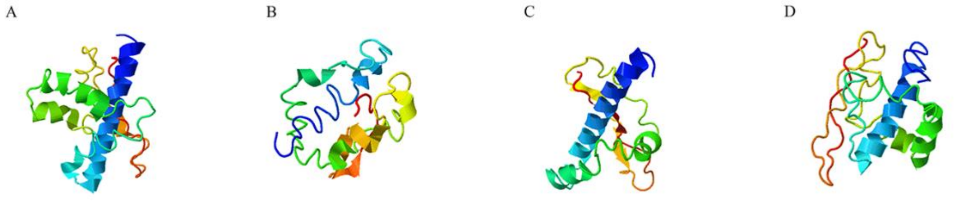

Supplemental Figure S1. 3D structure diagram of *PagSTOMAGEN* (A), *PtrSTOMAGEN* (B), *AtSTOMAGEN* (C), and *OsSTOMAGEN* (D). Prediction of 3D structures was done on the I-TASSER server.

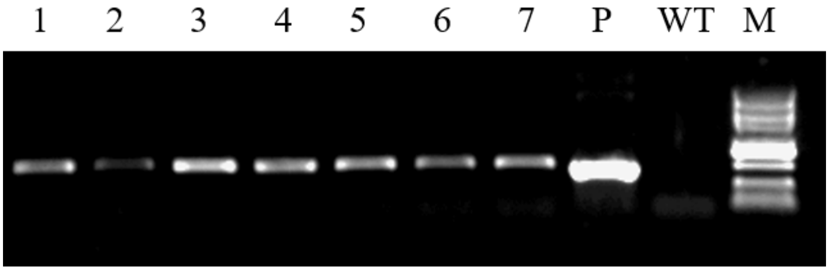

Supplemental Figure S2. Identification of transgenic poplar. P means positive control. WT means wild-type poplar. M means maker.

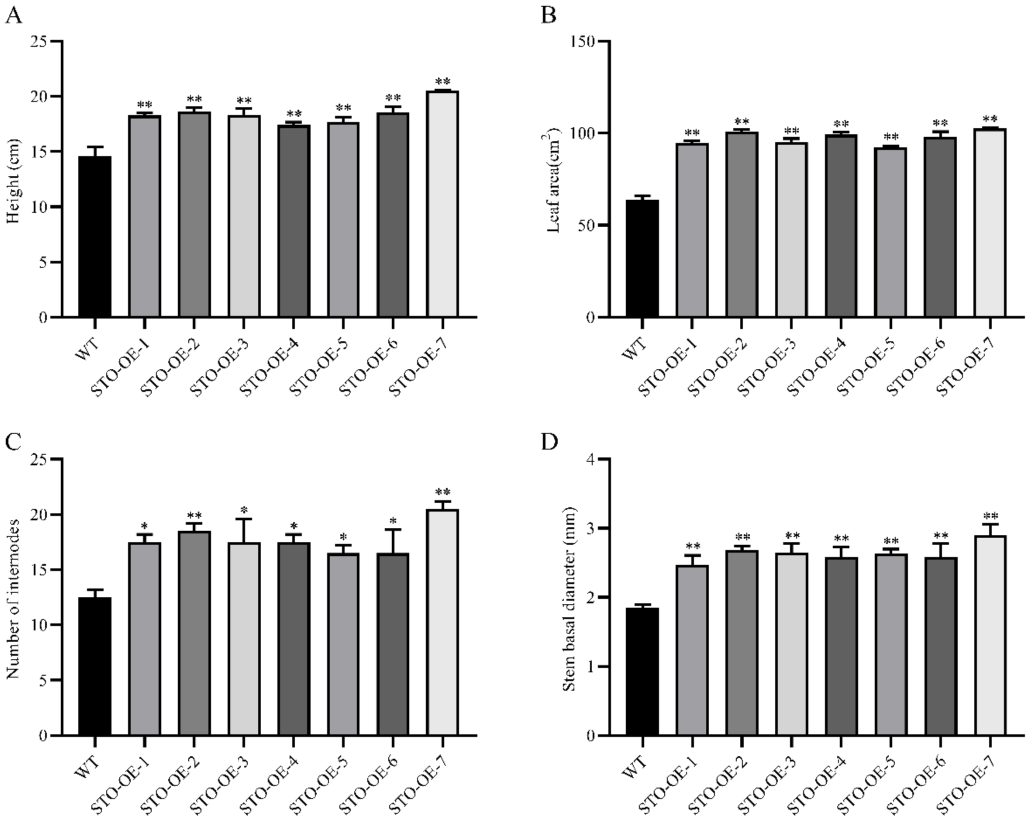

Supplemental Figure S3. Phenotypic measurements of *PagSTOMAGEN*-overexpressing (STO-OE) and wild-type (WT) lines.

(A) Statistical analysis of plant height of STO-OE and WT lines. (B) Statistical analysis of leaf area of STO-OE and WT lines. (C) Statistical analysis of the number of internodes of STO-OE and WT lines. (D) Statistical analysis of the stem basal diameter of STO-OE and WT lines. Data are presented as the mean ± SD. One-way ANOVA were used to determine variance among the mean values; **, $P < 0.01$; *, $P < 0.05$.

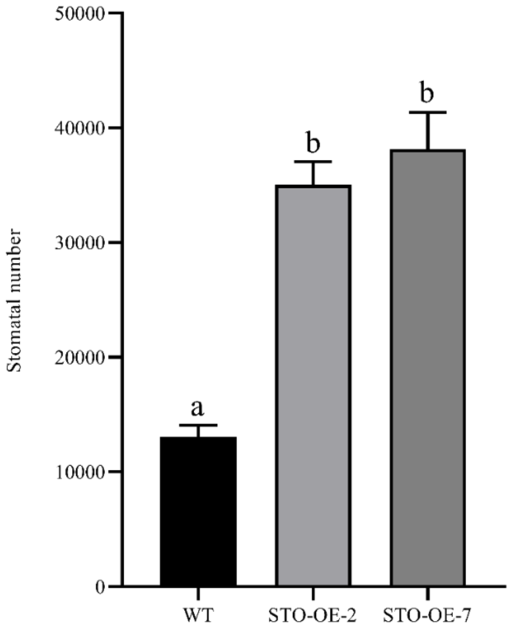

Supplemental Figure S4. Stomatal number of STO-OE and WT lines. Stomatal number = leaf area × stomatal density. Error bars represent the SD for each genotype with five plants. $P < 0.05$ was considered statistically significant and was represented by different letters.

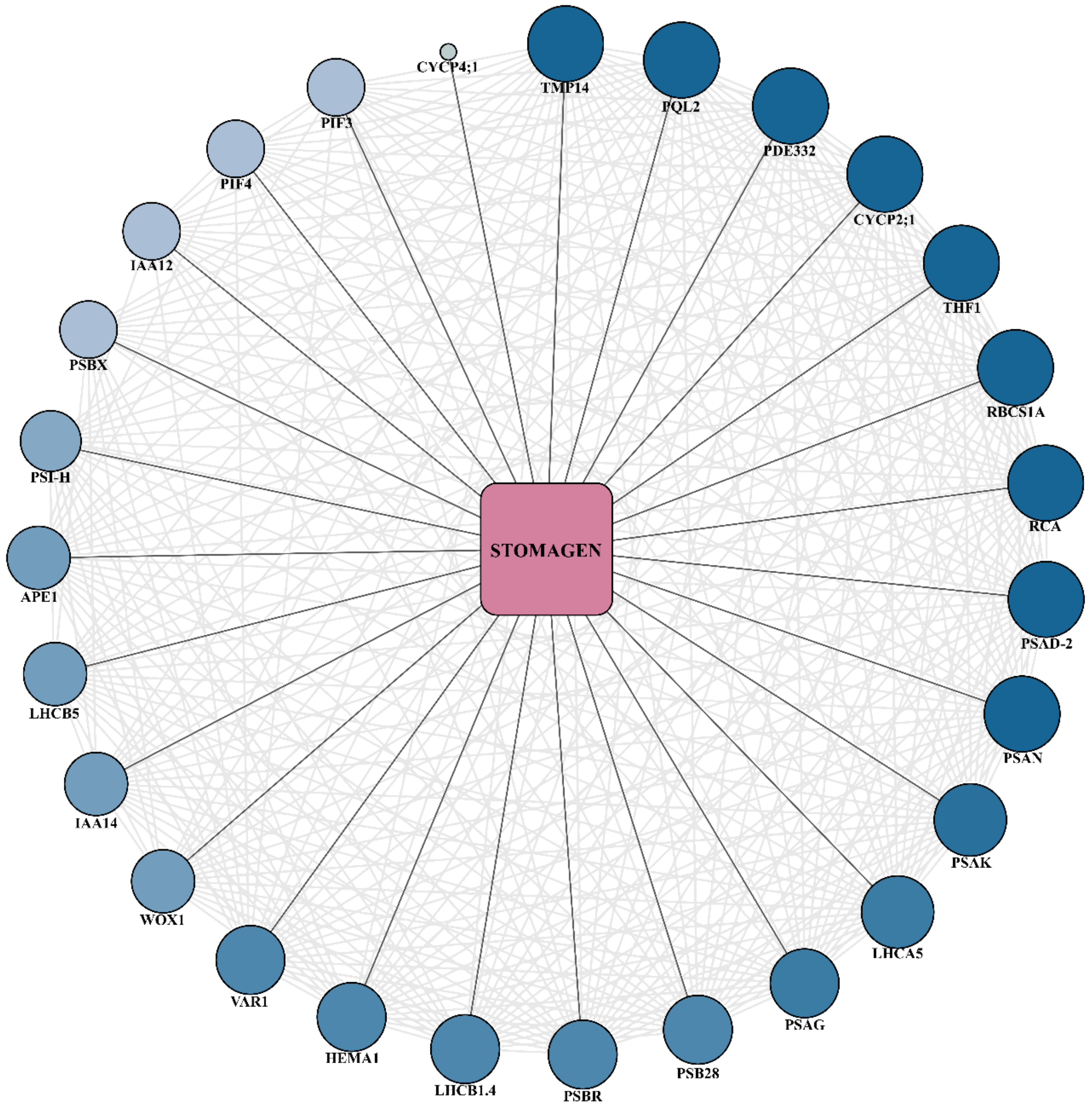

Supplemental Figure S5. Co-expression network centered on STOMAGEN. Blue circle nodes represent photosynthesis and growth-related genes. The node size is positively correlated with the gene degree.

Supplemental Table S1. Primers used for this research.

**Primer ID**	**Primer Sequence (5′->3′)**	Purpose
PagSTO-1F	ATGGCAAACACTAGACTATG	Cloning PagSTOMAGEN coding region
PagSTO-378R	TCACCTATGACAAATACATT	Cloning PagSTOMAGEN coding region
PagSTO-Q-13F	AGACTATGCTACTTACTATCCCTTC	qPCR of PagSTOMAGEN
PagSTO-Q-120R	AGAAGATAAGGTGGTCAGCG	qPCR of PagSTOMAGEN
ACTIN-Q-F	AAACTGTAATGGTCCTCCCTCCG	qPCR of ACTIN
ACTIN-Q-R	GCATCATCACAATCACTCTCCGA	qPCR of ACTIN
PBI121-PagSTO-1F	CGGGGGACTCTAATGGCAAACACTAGACTATG	Cloning PagSTOMAGEN coding region into PBI121 vector
PBI121-PagSTO-375R	ACTAGTCAGTCGACTCCTATGACAAATACATT	Cloning PagSTOMAGEN coding region into PBI121 vector
PagSDD1-Q-F	ACATAATTGCAGCCTGGCCT	qPCR of PagSDD1
PagSDD1-Q-R	ATGAGCTGAGCGGATGAGTG	qPCR of PagSDD1
PagTMM-Q-F	AATCCGATGGGGTCTGCTAC	qPCR of PagTMM
PagTMM-Q-R	GCCTAGTGACTCGGGGATTG	qPCR of PagTMM
PagERECTA-Q-F	GGCATTTCGTTTGGAACTTGTCT	qPCR of PagERECTA
PagERECTA-Q-R	AGACGATGGGGAGTCTGTCC	qPCR of PagERECTA
PagEFP1-Q-F	TGTCTTCCGACGGCCATTTC	qPCR of PagEFP1
PagEPF1-Q-R	GCTGGCCATCTCTCCCTGTA	qPCR of PagEFP1
PagEPF2-Q-F	GAAGTTCTTAGTTGGAGCCCACA	qPCR of PagEFP2
PagEPF2-Q-R	CTTGGGGAAATCATGAGCTTCTT	qPCR of PagEFP2
PagSPEECHLESS-Q-F	GGCGGTCAGTGTCACAAAAG	qPCR of PagSPEECHLESS
PagSPEECHLESS-Q-R	AGACATACGTTGCTGCCCAT	qPCR of PagSPEECHLESS
PagMUTE-Q-F	TAGGGCGCCACTGCAATTA	qPCR of PagMUTE
PagMUTE-Q-R	TCGCCGCGATATCACTTTCA	qPCR of PagMUTE
PagFAMA-Q-F	GCCTAGAATCCCAGAAGCGG	qPCR of PagFAMA
PagFAMA-Q-R	CTCAGCGGTTTCTTCACGGA	qPCR of PagFAMA

Supplemental Table S2. Amino acid sequences of the EPF/EPFL family.

>AtEPFL7
MDHVNPTLFHLKSLSIFTLTLLYISSPHFLLFKTLSMYENLRIFLKIIPFNLFGMKSV
SLIAILLLHLFVSSDTFQLDLF

HCKYYLAITWLINRDNECVIYILVGSFICCIVICEVVDKASGSSIPDCSNACGPCKP
CKLVVISSTCSASEACPLVYKCL
CKGKYYHVPSLT
>AtEPFL8
MDSSRKYKRCGFGAALFVANIFFSLLSLHCISGAHGHQQRMKESVMGSEPPVC
ATKCRNCKPCLPYLFDIRGAHDDDDDS
EPYYPVKWICRCRDRVFEP
>AtEPFL6
MGFERTSSSLSLLSSSLPSSLQPSENTRAKFSLFYLLLLFFVLCVIATFTITPTSTSSPY
NRNSNSGTLGNFYAKVRKLF
IPLTLLRDLTISDCDGMSRLTGHAEMPCTPKEILSRRIGFSAHEDKDSTQLLVRSP
LVGPCLAYDLDRVEEGKSTVVIKK
TRKIGDRSKEAELRRILRGLGSSPPRCSSKCGRCTPCKPVHVPVPPGTPVTAEYYP
EAWRCKCGNKLYMP
>AtEPFL3
MRVKETGKGFVVILMVMIPIVSWVDATSRPIASSHVSFYPEIIKPQAAEENSRRRI
LNPNENKEEIVKRRRRIGSKPPSC
EKKCYGCEPCEAIQFPTISSIPHLSPHYANYQPEGWRCHCPPP
>AtEPFL5
MGVVLPTLIVYAFLLFFSSSSAASLQRPSGGLGQGKKEIARSGLPGQIVDQKRLG
GPGSVPPMCRLKCGKCEPCKAVHVP
IQPGLIMPLEYYPEAWRCKCGNKLFMP
>AtEPFL9/STOMAGEN
MKHEMMNIKPRCITIFFLLFALLLGNYVVQASRPRSIENTVSLLPQVHLLNSRRR
HMIGSTAPTCTYNECRGCRYKCRAE
QVPVEGNDPINSAYHYRCVCHR
>AtEPFL4
MGTFRRRRRFLLAALVTFALLHLFSASSIVSADGRWIGQRTGSDLPGGFIRSNKR
FGGPGSSPPTCRSKCGKCQPCKPVH
VPIQPGLSMPLEYYPEAWRCKCGNKLFMP
>AtEPFL2
MVWSSNMSSFLLILLILNSTHFSLMANGRPEPDSVEFTKSGDQDVKMMMRGLIG
SRPPRCERVRCRSCGHCEAIQVPTNP
QTKLHSPLTTSSSSSSETIHLDYTRGDDSTNYKPMSWKCKCGNSIYNP
>AtEPFL1
MFAIYKSTLLLLPLILILLITPQVSSFLQPIQPPISPQVALIEDKARLGSTPPSCHNRC
NNCHPCMAIQVPTLPTRSRFT
RVNPFSGGFVRPPSSLTTVLDQYSNYKPMGWKCHCNGHFYNP
>AtEPF2
MTKFVRKYMFCLVLVFAACSLVVNSIRTPPLKNTVNGGEKKNADIEQAQTHHK
KEISKNGGVEMEMYPTGSSLPDCSYAC
GACSPCKRVMISFECSVAESCSVIYRCTCRGRYYHVPSRA
>AtEPF1

MKSLLLLAFFLSFFFGSLLARHLPTSSHPSHHHVGMTGALKRQRRRPDTVQVAG
SRLPDCSHACGSCSPCRLVMVSFVCA
SVEEAETCPMAYKCMCNNKSYPVP
>PagEPFL7
AMKHPLFYVLAIFVAAALIIPLMTMSAVRVSRHSSRERYTTWPRKRNSTVGRLTL
TSKGVARERMKQNGAHPLQIAGSRLPDCSHACGSCTPCVLKIVSSPCSSLAQSE
ACPISYKCMCNNKYYPVP*
>PagEPFL10
AMKHPLFYVLAIFVAAALIIPLMTMSAVRISRHSSRERYTTWPRKRNSTVGRLTL
TSKGVARERMKQNGAHPLQIAGSRLPDCSHACGSCTPCVLKIVSSPCSSLAQSE
ACPISYKCMCNNKYYPVP*
>PagEPFL1
MKVFVAASVIIALFLLPMAISARHIGRPRTHHGHHHSTQPRIKDGTVAKPKFLY
GERWPAKRRGADTVQIAGSSLPDCSHACGSCSPCRLVMVSFVCASLEEAETCP
MAYKCMCNNKSYPVP*
>PagEPF1
SLLKEKSNTTPLKLTKAMKIFVAALVIALICLPTAISARHIGRPRTHHGHHHSTQ
PRIKDGTVAQPNFYRERWPAKRRGDTVQIAGSSLPDCSHACGSCSPCRLVMVSF
ICASLEEAETCPMAYKCMCHNKSYPVP*
>PagEPFL2
MKFLVGAHKSLLLMLIFMVFMVGKSLRPHHYTKSSYSKEGASNGEEAHDFPKE
AAREELGMELYPTGSSLPDCSHACGPCTPCKRVMVSFKCSVAESCPIVYRCMCK
GKYYHVPSA*
>PagEPF2
MKFLVGAHKSLLVMLIFMVFMVGKSLRPHHYTSPATQRRSVQMGMRSISPKGS
RRRARNGTISAISLPTALMPVDLALHARGCMCKGKYYHVPSA*
>PagEPFL4
MGSCHLLPASRSLLTTAISIYFLLYPVSCFDQHLQPPTSPHKGIAFEGKARLGSTPP
SCRSKCNGCHPCIAVQVPALPSQNQPVQVGSAKRTRSVDEFFDSYPAGNRFPDY
RPLGWKCRCADHFYNPLT*
>PagEPFL5
MGSCLLLPASRSLLTTAISIYFLLYPVSCFDQHLQPPTSPHKGIAFEGKARLGSTPP
SCHSKCNGCHPCIAVQVPALPGQNQPVRVGSAKRTRSVDEFFDSYPAGNRFPD
YRPLGWKCRCADHFYNPLT*
>PagEPFL6
MATASCATLLLHFLLSTVFCFAQHDLQPPIASHEGAAFEEKARLGSTPPSCHNK
CNGCHPCMAVQVPTLPNPNRPAQPVSTKTSIIDPFFDPYPAGNNRTVSIENPEC
TKNMPEKLKTINIFIKLVASWQCKIYSICLCLT*
>PagEPFL3
MGSAKIGFSCHRNRQLIISLLFILVSSSTLVRFTAEGRATTKLLEAAPEKGVEEEKI
AVRAQTIGSRPPRCDKRCTSCEHCEAVQVPITTQAHSRKRSRFSAAISNIAYSRG
DGISNYKPMSWKCKCGNLIFNP*
>PagEPFL11

MEAGSICCLVLALHTVSLVSAASRPFAPNNGVAVNQAVTGHSLQSLQVPLDSN
FDPQSGSEKGIKTKDGVAGGMIIEAAAEVTLKALQKLGPPHQVVCTSAMAALL
VKQFKCPPSAKPAPTI*
>PagEPFL12
MTLLRHRHHFLSTLTFFTFLLFLSAPATTLSQLGSGVLQQGGEGRGKGGGLRAF
QRVLTQKRLGGPGSSPPSCRSKCGKCSPCKAVHVAIQPGLSMPLEYYPEAWRCK
CGNELFMP*
>PagEPFL13
MSLLRPRHHLSTLTAFTFLLFFSASATTVLLSQPGSGSLQEGGEDKGKQSGLGAF
PRVLTQKRHGGPGSSPPSCRSKCDKCSPCEPVHVPIQRGWRMPLEYYPEAWRC
KCGNKLFMP*
>PagEPFL14
MECLKRKRSLIFNNKPSLFHYVSFTIFFFFVSFVALFSPFTSNTTCLGMRCPLSEKA
DNFYFQEFEKTRSGYDEQVEVSSSMARRFLSGPGSSPPRCTSKCGNCTPCKPVHV
AMPPGTPVTTEYYPEAWRCKCGNKLYMP*
>PagEPFL15
MELKSKRSLIFFNKEPSLFQCVSFTIFFILSFVALFSTISSKTTCLGIIRCPLSGKARDF
YFQEFEKTRGYEGQMEASLSMARRFLSGPGSSPPRCTSKCGKCTPCKPVHVPVPP
GTPVTAEYYPEAWRCKCGNKLYMP*
>PagEPFL8
MASATDSSHGIRVTFIVILFLSLTFLPPVSVGSSTPSRGSEDMKQKKMVLGSRPPQ
CVNRCSNCKPCMAALVTPPHHKNGVRGPSSSKGDESYYLLSWKCKCGDKYFQ
P*
>PagEPFL9
MANTRLCYLLSLLFTFILAATAIFSFLFGCRENTLTTLSSSFDPEIKSCCLIIKVSVH
HHRKDSQALGGNEEQMSSKRLMIGSTAPTCTYNECRGCKYKCRAEQVPVEGN
DPIHSAYHYKCICHR*XXXXXXXXXXXXXX
>OsEPF1
MWHVPSRPESPAFNLCPHFVHYVRTTFLCIRNAAGVQGSAGGLHNGGTAPSA
AAANGSTTTAYDDRGTGGQTATFQVQQGAQPEEETTTEMGNAAEAATGSRLP
DCTHACGPCSPCRRVMVSLRCAEAAESCPVAYRCMCRGRFFRVPTL*
>OsEPF2
MRRHATLADARGHHRILLILAVVLLLAATGDGIRSPPYDVVTPVHGAQDEKISA
TATLVISPQQATAGDNVGRGVLQEEVRATGSSLPDCSHACGACSPCNRVMVSF
KCSIAEPCPMVYRCMCKGKCYPVPSS*
>OsEPFL1
MRTAATPPLAAAAAAVAAVFLSALLLASASASASRLPPPRRLLPLVGGEVAVA
VVAGEEEKVRLGSSPPSCYSKCYGCSPCVAVQVPTLSAPSVPAAAAPRTTPRRS
WRRSPTTSR*
>OsEPFL2
MAVRSPHRAFLVAMILVSFLLGAAAGIRSTTLSSSQSLAEDKSRLGSTPPSCHNR
CNACNPCTPVQVAALPGVSRPASAADRVDVAGFAQYSNYKPLGWKCRCAGR
LFDP*
>OsEPFL3

ISLNSDAAITSAAAAGGELHHHQIKEPPPETGGKGWAAMSEALIGSRPPRCEGK
CAPCGRCEAVQVPVAPRAGRLRAFFFSRAAAADDDDESSTNYKPLNWKCRCA
DTRRALDP*
>OsEPFL4
TGGRTTTTTTTGRDGCWLVPPFFASSHPVATQLLPPKSSSKWRAICSCSSCSLSSS
PPPSLPKLPPASPRSEKGVAGIRGVIGSRPPSCEGRCRSCGHCEAVQVPITPQQLQ
KKRGQGDDRRRKKKQLLGHGDRAAAAGGRAMPDSYDDHSNYKPLSWRCKC
GGMILNP*
>OsEPFL5
MVHLLPCTHAHLPLPLFFSILLLIFSMEVAHSDARRLPLKLLEVGNIKEEPDETIG
EKMEMEMEGRRLIGSRPPRCERVCMSCGHCEAVQVPIVPQVIQKTQTKAAAAA
AAEQEQHVVVSATAISAAVFTYRVNGLSNYKPLSWKCKCGGIILDP*
>OsEPFL6
MASARRGRRRRVPVAASPLLILLVFLLAASLGACSSARKRSGDGGGAVAEEVYY
SSWGSAVAVAGRRRLVGPGSSPPTCRSRCGGCHPCRPVHVAIQPGRSFPLEYYP
EAWRCKCGDKLFMP*
>OsEPFL7
MHATVAAAARRPIRVLRRPAAAAAHVRRLDSSSSLMLARRTNAAAGGGDNG
DVRRRRLIGPGSSPPTCRARCGRCAPCRPVHVAIQPGVGAQWEYYPEVWRCKC
GDKLFMP*
>OsEPFL8
MMPACGMGSSRRPRRWSGGSKLAVACLAAVAVTSLQLCCLSGCFIAACGGAG
RDDDDVRRYSDHFGRLEGAGAHSGDLLEGGGHYHVGLGRRLLSGGPGSHPPR
CTSKCGSCSPCSPVHVSVPPGVLVTTEYYPEAWRCKCRNRLYMP*
>OsEPFL10
MASSGGRWRRSGSSMLKLVVALCFFAVAISLCCVCLSSGCGGSGCGGFRRRAVL
LRLGNEIQRAASFCALDHSSSGRRWRRLLAEGPGSYPPRCTAKCGACVPCYPVH
VAVPPGVPVTTEYYPEAWRCKCGNRLYMP*
>OsEPFL9
MANACPTSTTSSLPLFFLFCFLLFSHARCNQGHHGSISGTDYGEQYPHQTLPEEH
IHLQENIKVLNKERLPKYARRMLIGSTAPICTYNECRGCRFKCTAEQVPVDAND
PMNSAYHYKCVCHR*
>PtEPFL7
MSRHFINYRTNRILTHGNIILYRGTQDAQAIQCGRRIHVRTLGSPFSWLNNFTKL
NPRAMKHPLFYVLAIFVAAALIIPLMTMSAVRISRHSNRERYTTWPRKRNSTMG
RLTLTSKGVVRERMKQNGAHPLQIAGSRLPDCSHACGSCTPCVLKIVSSLCSSLA
QSEACPISYKCMCKNKYYPVP*
>PtEPFL1
MRKTKAMKIFVAALVIIALCLLPMAISARHIGRPRTHHGHHHSTQPRIKDGTVA
KPNFLYRERWPEKRRGADTVQIAGSSLPDCSHACGSCSPCRLVMVSFICASLEEA
ETCPMAYKCMCDNKSYPVP*
>PtEPF1

MKIFVATLVIALICLPTAISARHIGRPRTHHGHHHSTQPRIKDGTVAQPNFYRER
WPAKRRGDTVQIAGSSLPDCSHACGSCSPCRLVMVSFICASLEEAETCPMAYKC
MCHNKSYPVP*
>PtEPF2
MKFLVGAHKSLLLMLIFMVFMVGKSLRPHHYTKSSYSKEGASNGDEAHDFPKE
AAREELGMELYPTGSSLPDCSHACGPCTPCKRVMVSFKCSVAESCPIVYRCMCK
GKYYHVPSA*
>PtEPFL4
MGSCRLLPASRSLVLTTAISIYFLLYPVSCFDQHLQPPTSPRKGIAFEEKARLGSTP
PSCHNKCNGCHPCIAVQVPALPSQNEPVQMGSAKTSSIDEFFDSYPAGNRFPDY
RPLGWKCRCADHFYNPLT*
>PtEPFL5
MGSYHQCFLFSATVLLHILLSTVFCFAQHDLQPPIASHEGAAFEEKARLGSTPPS
CHNKCNGCHPCMAVQVPTLPNQNRPAQPVSTKTSIIDPFFDPYPAGNNRYSNY
KPLGWKCRCGDHFYNPLS*
>PtEPFL3
MGSAKIGFSCHRNRQLIISLLFILVSSSTLVRFTAEGRVTTKLLEAAPEKGVEEEKI
AVRARTIGSRPPRCDKRCNSCEHCEAVQVPITTQAHSRKRSRFSAAISNIAYSRG
DGISNYKPMSWKCKCGNLIFNP*
>PtEPFL6
MEASSICCLVLALQIVSLVSAASRPFAPNSGVAVNRAVTGHSLQSLQVPLDSNF
DPQSGSEKGIKTKDGVAGGMIIEAAAGYAKGITKIGSTPPSCEHKCHGCTPCEAI
QVPAISKTGTHHLSVNYANYEPEGWKCKCGPSFYSP*
>PtEPFL8
MTLLRHRHHFLSTLTFFTFLLFLSAPATTLSQLGSGVLQQGGEERGKGGGLRAF
QRVLTQKRLGGPGSSPPSCRSKCGKCSPCKAVHVAIQPGLSMPLEYYPEAWRCK
CGNELFMP*
>PtEPFL10
MSLLRPRHHLSTLTAFTFLVFFSASATTVLLSQLGSGSLQEGGEDKGKGSGLGAF
PRVLTQKRHGGPGSSPPSCRSKCDKCSPCEPVHVPIQHGWRMPLEYYPEAWRC
KCGNKLFMP*
>PtEPFL11
MECLKRKRSLIFNNKPSLFHYVSFTIFFFFLSFVALFSPFTSNTTCLGIRCPLSEKAD
HFYFQEFEKTRGYDEQMEVSSSMARRFLSGPGSSPPRCTSKCGNCTPCKPVHVA
MPPGTPVTTEYYPEAWRCKCGNKLYMP*
>PtEPFL12
MELKSKRSLIFFNKEPSLFQCVSFTIFFFLSFVALFSTISSKTTCLGIIRCPLSGKARN
FYFQEFEKTRGYGDPMEVSLSMARRFLSGPGSSPPRCTSKCGKCTPCKPVHVPVP
PGTPVTAEYYPEAWRCKCGNKLYMP*
>PtEPFL2
MASATDSSYGIRITFIVILFFSLTFLPPVSAGSSMPSRGSEDMKQKKMVLGSRPPQC
VNRCSNCKPCMAALVTPPHHKNGVRGPSSSKGDESYYLLSWKCKCGDKYFQP*
>PtEPFL9

MANTRLCYLLSLLFTFILAAFVIQGSRNQELLPYHQSISTPSQEDSQALGGNEEQ
MSSKRLMIGSTAPTCTYNECRGCKYKCRAEQVPVEGNDPIHSAYHYKCICHR*

Supplemental Table S3. DEGs in STO-OE and WT poplars.

Supplemental Table S4. The number of DEGs in STO-OE and WT poplars.

Supplemental Table S5. GO terms of DEGs between STO-OE and WT lines in the 1st leaf position.

Supplemental Table S6. GO terms of DEGs between STO-OE and WT lines in the 5th leaf position.

Supplemental Table S7. GO terms of DEGs between STO-OE and WT lines in the 10th leaf position.

Supplemental Table S8. GO terms of DEGs between STO-OE and WT lines in young stem segments.

Supplemental Table S9. DEGs involved in the photosynthesis and growth in the 1st leaf position.

Supplemental Table S10. DEGs involved in the photosynthesis and growth in the 5th leaf position.

Supplemental Table S11. DEGs involved in the photosynthesis and growth in the 10th leaf position.

Supplemental Table S12. DEGs involved in the photosynthesis and growth in

young stem segments.
