# Supplementary figures and images for: Overexpression of PagSTOMAGEN, a Positive Regulator of Stomatal Density, Promotes Vegetative Growth in Poplar
Source: Int J Mol Sci. 2022 Sep 5;23(17):10165. doi: 10.3390/ijms231710165 (PMC9456429; doi:10.3390/ijms231710165)

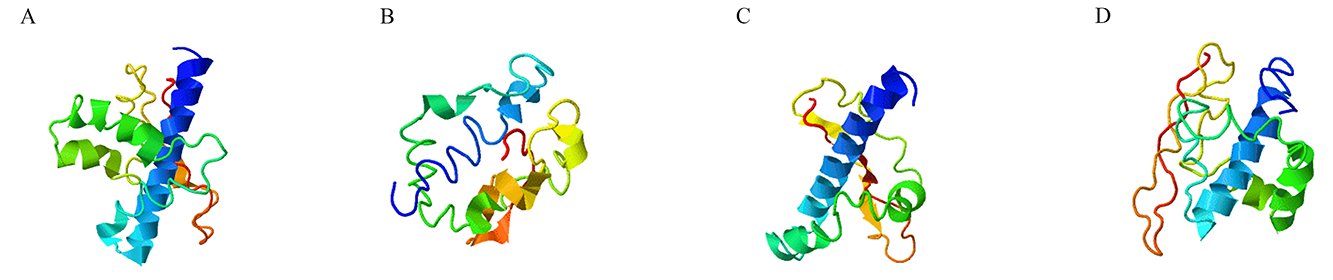

Supplement: Supplementary file 1 [file ijms-23-10165-s001.zip › ijms-1892419-supplementary/Figure S1.tif]

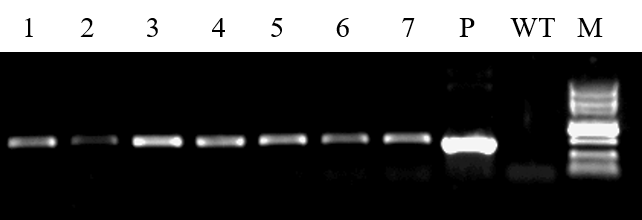

Supplement: Supplementary file 1 [file ijms-23-10165-s001.zip › ijms-1892419-supplementary/Figure S2.tif]

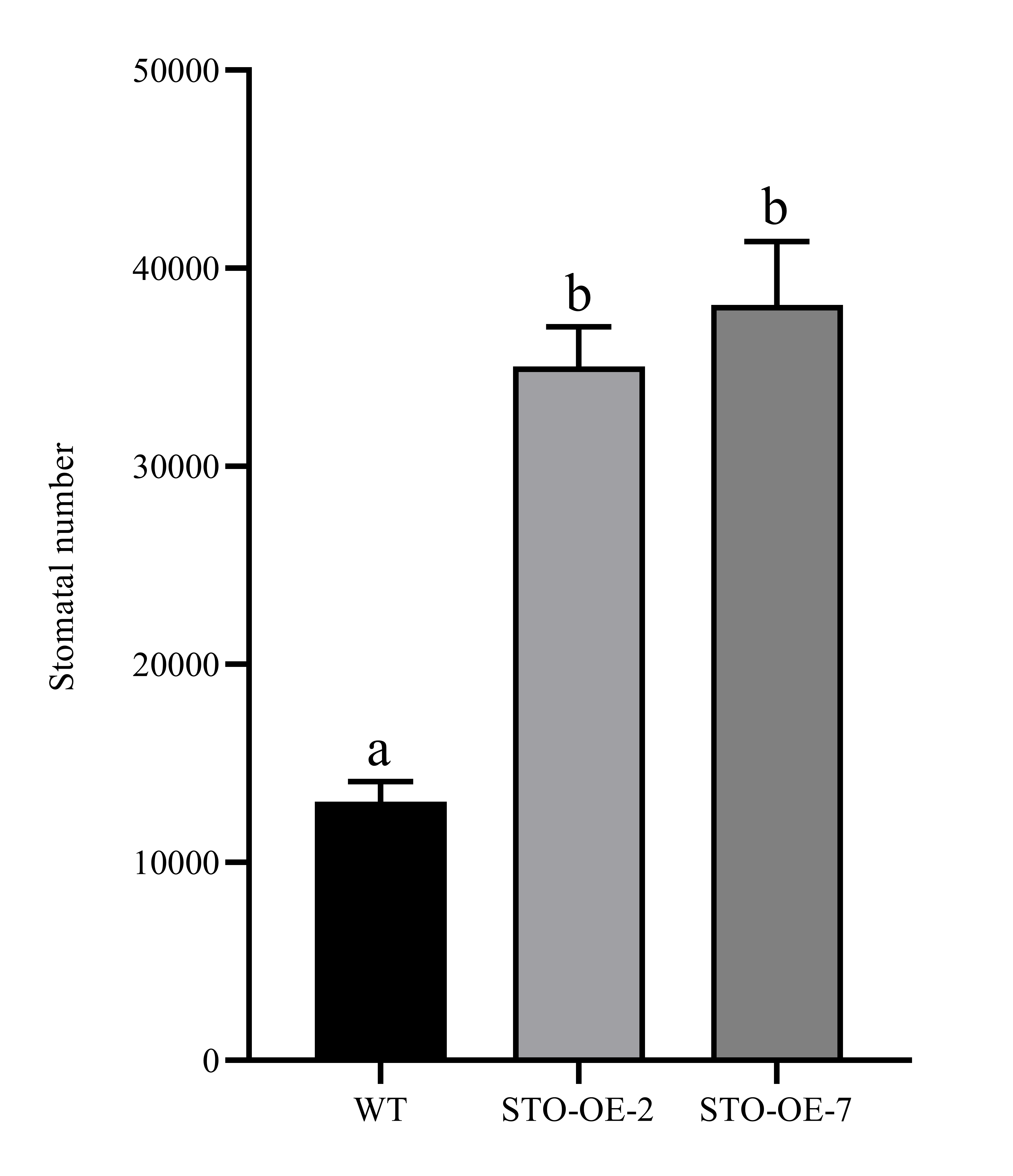

Supplement: Supplementary file 1 [file ijms-23-10165-s001.zip › ijms-1892419-supplementary/Figure S4.tif]
